# Two behavioural traits promote fine-scale species segregation and moderate hybridisation in a recovering sympatric fur seal population

**DOI:** 10.1186/1471-2148-10-143

**Published:** 2010-05-14

**Authors:** Melanie L Lancaster, Simon D Goldsworthy, Paul Sunnucks

**Affiliations:** 1 Zoology Department, La Trobe University, Bundoora, Victoria 3083, Australia; 2 School of Earth and Environmental Sciences, The University of Adelaide, Adelaide, South Australia 5005, Australia; 3 South Australian Research and Development Institute, South Australia 5024, Australia; 4 School of Biological Sciences and Australian Centre for Biodiversity, Monash University, Clayton, Victoria 3800, Australia

## Abstract

**Background:**

In systems where two or more species experience secondary contact, behavioural factors that regulate interspecific gene flow may be important for maintaining species boundaries and reducing the incidence of hybridisation. At subantarctic Macquarie Island, two species of fur seal breed in close proximity to one another, hybridise at very high levels (up to 21% of hybrid pups are born annually), yet retain discrete gene pools. Using spatial and genetic information collected for pups and adults over twelve years, we assessed two behavioural traits - inter-annual site fidelity and differences in habitat use between the species - as possible contributors to the maintenance of this species segregation. Further, we explored the breakdown of these traits in pure-species individuals and hybrids.

**Results:**

We found virtually complete spatial segregation of the parental species, with only one exception; a single territory that contained adults of both species and also the highest concentration of hybrid pups. The spatial distribution of each species was closely linked to habitat type (pebbled vs boulder beaches), with members of each species breeding almost exclusively on one type or the other but hybrids breeding on both or at the junction between habitats. Inter-annual site fidelity was high for both sexes of pure-species adults, with 66% of females and all males returning to the same territory or a neighbouring one in different years. An important consequence for pure females of breeding on the 'wrong' habitat type, and thus in a heterospecific aggregation, was the production of hybrid pups. Low habitat fidelity of hybrid females facilitated bi-directional backcrossing, resulting in more diverse hybrid offspring.

**Conclusion:**

In a disturbed system where two sympatric fur seal species breed in close proximity, discrete gene pools are retained by extremely fine-scale and strong spatial segregation of the species. Two behavioural traits were found to be important in maintaining this stable population structure, and habitat type was a strong indicator of where species locate and a potentially powerful predictor of future directions of hybridisation. A direct consequence of the breakdown of this trait was the production of hybrid offspring, which may have severe implications if hybrids have reduced fitness.

## Background

Secondary contact between previously isolated species can result in hybridisation unless mechanisms are in place to prevent it. Outcomes of this depend on the fitness of hybrids relative to parental species, as well as behavioural and ecological attributes that contribute to the maintenance of species boundaries [1,2]. In systems where human-induced disturbance has resulted in sympatry, exploring factors that influence interspecific gene flow is important for understanding population genetic structure, past evolutionary processes and future trajectories of hybridisation.

In sympatry, species-specific differences in ecology, behaviour or physiology may allow discrete gene pools to be maintained. Adaptation to different habitats can reduce interspecific encounter rates and subsequent gene flow, thereby forming a barrier to hybridisation [3]. For example, different habitat use as a result of dietary specialisation appears to be important for maintaining spatial separation and species boundaries in several sympatric terrestrial mammals, including mice, wallabies, chipmunks, voles and shrews [4-6]. In sympatric phytophagous insects, host plant specificity is thought to inhibit hybridisation and even promote speciation [e.g. [7-9]]. Behavioural traits such as recognition of conspecifics and mate choice can also help to shape the distribution of species by reducing heterospecific encounters and promoting the formation of single-species aggregations [1,2]. Separation of species may be further enhanced in populations that have stable structuring over time, such as those where individuals display mating site fidelity or natal site philopatry.

Colonially-breeding pinnipeds (seals) utilise the marine environment for foraging but return to land to breed, moult, and provision their young. This partitioning of habitats means that spatial separation of sympatric species at sea (e.g., benthic vs pelagic foraging in Australian and New Zealand fur seals, *Arctocephalus pusillus doriferus *and *A. forsteri*, [10]) does not, by default, lead to their spatial separation on land. Suitable breeding habitats for land-breeding seals are limited to small islands, rocky outcrops, and coastlines. Generic criteria for specific mating and birthing sites are thought to include proximity to water and foraging grounds, safety from predators and shelter from inundation during high seas [11]. Any spatial segregation of sympatric species on land would presumably result from additional, fine-scale requirements or preferences. These may be related to physical habitat characteristics (e.g., shade, tide pools, rock type [12]) or behavioural traits such as conspecific recognition or mate choice. Intra-specific differences in habitat use by pinnipeds according to age and breeding status have been observed [13], but there have been few observations of sympatric seals being consistently segregated by particular habitats, and if so, what the reasons behind this may be.

A small population of sympatric fur seals at subantarctic Macquarie Island forms an excellent free-living system in which to explore how habitat use and behavioural components may affect interspecific gene flow and hybridisation. Antarctic (*Arctocephalus gazella*) and subantarctic fur seals (*A. tropicalis*) experienced severe over-harvesting from the 18th to the early 20th century [14] but are now recovering, with global population sizes of *A. gazella *in the millions and *A. tropicalis *approximately 300,000 [15,16]. *Arctocephalus gazella *generally breeds south of the Antarctic Polar Front (APF) while *A. tropicalis *breeds north of the APF, but the species are sympatric at three sites: Îles Crozet, Marion Island and Macquarie Island (where males of a third species, New Zealand fur seals, *A. forsteri*, also occur). Levels of hybridisation at Marion and Amsterdam islands are low (~2% [16,17], but recent genetic analysis of the breeding population at Macquarie Island revealed up to 21% of pups born to be *gazella/tropicalis *hybrids [18].

Differences in the timing of recolonisation by both sexes of *A. gazella *and *A. tropicalis *have most probably contributed to the high levels of hybridisation at Macquarie Island [19]. Subsequent establishment of breeding populations of both species has resulted in an overall decline in hybridisation, and neutral genetic markers indicate that both pure lineages are maintained in the population [18,20]. The fitness of hybrid offspring is yet to be fully explored but post-F_1 _hybrids and backcrossed individuals occur in the population and most types of hybrids have successfully produced pups, indicating some degree of viability and fertility [18,21]. A reproductive cost of hybridisation has been identified through mating avoidance of hybrid males by females [21,22], however, continued production of hybrid offspring indicates that females may differ in the level of assortative mating they exercise (also observed in [23-25]). Thus, while female mate choice is an important component of the system, analysis of other factors that may be influencing hybridisation is instructive for understanding its outcomes in the population.

At Macquarie Island, the requirements for breeding sites are expected to be similar for *A. gazella *and *A. tropicalis*. However, fine-grain, species-specific differences in habitat use have been observed across the allopatric distributions of the two species: *A. gazella *favours open, shingled beaches while *A. tropicalis *typically utilises irregular rock platforms or boulder beaches [26,27]. This pattern is consistent at the three sympatric locations [16,19,28]. The reason for this difference is unclear but may represent strongly programmed preferences, because it is observed even at Macquarie Island, where the breeding population is only a few percent of pre-sealing carrying-capacity and competition for space is minimal (~200 pups born annually [19]). Evidence of mate choice in the population suggests that the two species can recognise each other and may preferentially form conspecific aggregations. This segregation may be enhanced not only by different habitat use, but also by mating site fidelity, which has been previously demonstrated in *A. gazella *and other pinnipeds [29-31], and may stabilise the population structure over time.

At locations where *A. gazella *and *A. tropicalis *are sympatric, spatial segregation of the two species is likely to restrain heterospecific encounters and subsequent hybridisation. If hybrids have substantially lower fitness than parental species, factors that maintain this segregation, such as differing habitat preferences and high inter-annual site fidelity may also be under strong selection. Since both parental species and *gazella/tropicalis *hybrids breed on Macquarie Island, we were able to combine spatial and genetic data collected over twelve years to 1) explore the distribution of *A. gazella *and *A. tropicalis *and their hybrids within the population, 2) quantify species-specific habitat use and among-year site fidelity of breeding adults to determine whether these factors are likely to maintain segregation between parental species, and 3) assess the reproductive consequences for pure and hybrid adult females of low site fidelity and less constrained habitat use.

## Methods

### Study site, sampling and observational data collection

Macquarie Island (54° 30' S, 158° 56' E) is situated in the Southern Ocean, approximately 1500 km south-east of Australia. The island contains breeding populations of *A. gazella *and *A. tropicalis*, and a large transient group of male New Zealand fur seals, *A. forsteri*. As male *A. forsteri *do not frequent breeding areas they were thought not to be involved in mating. However, genetic analysis of hybridisation has identified *A. forsteri *hybrid pups in the population [18]. This was the result of a small number of *A. forsteri *hybrid males holding territories over multiple years as well as a small proportion of females mating away from breeding beaches with pure *A. forsteri *males [25]. Due to the absence of a breeding population of *A. forsteri *on Macquarie Island, their spatial distribution and habitat use was not explored. *A. forsteri *hybrids (pups, mothers and territory males) were excluded from all analyses.

**Figure 1:**
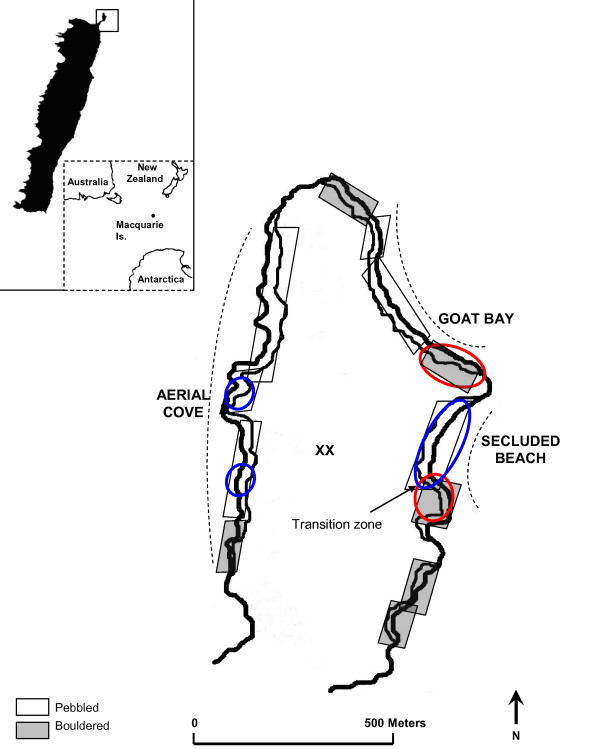
**Fur seal breeding beaches on North Head Peninsula, Macquarie Island, illustrating typical *A. gazella *habitat (open pebbled beaches, open boxes), *A. tropicalis *habitat (bouldered coves, grey boxes) and the main territory locations from 1992-2003 (blue circles, *A. gazella*, red circles, *A. tropicalis*)**. X indicates unsuitable habitat for fur seals and dashed lines mark the boundaries of each bay.

The fur seal breeding system is described as resource-defence polygyny, where males arrive before females and establish discrete territories [11]. Once on the beach, females give birth, mate, and within approximately ten days leave territories temporarily to forage at sea, after which time they alternate foraging with onshore nursing until their pup is weaned [11]. Breeding on Macquarie Island occurs annually from November to March on the northern tip of the island, North Head Peninsula, in three bays: Secluded Beach, Aerial Cove and Goat Bay. Each bay consists of two beach types: open, pebbled beaches (typically favoured by *A. gazella*), and bouldered coves with steep, overhanging cliffs (typically favoured by *A. tropicalis*, Figure 1). All bays are readily accessible to fur seals, and individuals (non-breeding adults, subadults and breeding females with pups older than two months of age) are often observed moving between bays during the summer months. The main breeding aggregations are formed on pebbled beaches in Aerial Cove and Secluded Beach (excluding the southern end), and bouldered coves in Goat Bay (Figure 1). At the southern end of Secluded Beach there is a 10 m north-south transition zone from pebbles to boulders (Figure 1). Differences in rock size in all breeding bays were confirmed by counting the number of rocks within 1 m^2 ^quadrats randomly selected across all areas encompassing breeding fur seals.

Eight complete cohorts of pups (1007 individuals) were sampled between 1992 and 2003 [18]. Adult fur seals present on breeding beaches were sampled opportunistically over the twelve year period and females were identified among years by tags placed in the trailing edge of fore-flippers (Dalton Size 1 Supertags, Table 1). Fewer adult males were tagged over the study period but some were identifiable among years by characteristic natural markings. Sampling of males was largely restricted to territory males and nearby challengers and was less comprehensive from 1999-2003, with approximately 20% of territory males sampled [21]. Breeding territories were mapped over all years and daily observations of the colony provided the territory locations of adult females at the time of parturition (birth) and mating, territory males during the mating season and birth locations of pups (Table 1).

**Table 1:** Numbers of *A. gazella*, *A. tropicalis *and hybrid individuals with known birth or breeding locations included in all analyses.

Age/sex class	***A. gazella***	***A. tropicalis***	Hybrid
Pups	597	173	135
Breeding females			
Single year observations	183	24	22
Multiple year observations (no. records)	95 (303)	12 (37)	9 (23)
Territory males			
Single year observations	13	10	4
Multiple year observations (no. records)	8 (20)	6 (15)	4 (12)

Pups represent eight complete cohorts from 1992-2003 and adults represent opportunistic sampling across that time period.

### Genetic analysis and species identification

Genotypes and species assignments of *A. gazella, A. tropicalis *and hybrid individuals from [18] were used in this study. For each individual this comprised a species-specific mitochondrial DNA (mtDNA) RFLP profile from a 417 bp fragment of the tRNA^thr^- control region, a nine-locus microsatellite genotype, and subsequent posterior probabilities of membership to each of the two species (*Q *values), obtained from the software program STRUCTURE [32]. Pure *A. gazella *individuals had mtDNA profiles and *Q *values ≥ 0.9 for belonging to that species (*Q*_*Gaz*_), pure *A. tropicalis *individuals had mtDNA profiles specific to the species, low *Q*_*Gaz *_values (<0.1) but high *Q*_*Trop *_values (≥0.9), and hybrids had *Q *values of <0.9 for belonging to either *A. gazella *or *A. tropicalis*, i.e. their microsatellite genotypes contained genetic characteristics of both species. A small number of pups (n = 4) had an *A. tropicalis *mtDNA profile but a microsatellite genotype typical of *A. gazella*. These pups were classed as post F_3 _backcrosses. Previously defined and validated ranges of *Q *for first generation (F_1_) hybrids and post-F_1 _hybrids were used to differentiate between hybrid classes (*Q *of F_1 _hybrids ranges from 0.22-0.78 [18]).

### Species distributions in relation to habitat type

Using territory locations of individuals and the genetic coefficient *Q*_*Gaz*_, we explored the spatial distribution of pure species to determine how segregated were breeding populations of *A. gazella *and *A. tropicalis *and whether the trend of different habitat use observed in other colonies was the same at Macquarie Island. We also plotted the spatial distribution of hybrid adults and pups to explore their habitat use and identify important sites of hybrid pup production. For hybrid adults, as well as using the genetic coefficient *Q*_*Gaz *_(derived from their microsatellite multi-locus genotypes), we examined habitat use in relation to their mitochondrial DNA profile to determine whether their matrilineal background influenced habitat use.

### Inter-annual site fidelity

The distance an individual moved between years was calculated as the distance from one territory boundary to another, and was accurate to within a few metres since territory areas were generally no greater than 15 m in diameter. For adult females and territory-holding males with known breeding locations over two or more years, site fidelity was analysed based on the 1) number of territories an individual occupied over that time, 2) maximum number and proportion of years spent in the same territory, and 3) maximum distance moved among years.

### Consequences of reduced site fidelity and less discriminating habitat use

If species-specific habitat use maintains species segregation and reduces heterospecific encounters and hybridisation, a measurable outcome of breeding in a heterospecific habitat for an adult female would be the species/hybrid status of her resulting offspring. Offspring *Q *values of pure-species females that bred in conspecific aggregations were compared with those that bred in heterospecific aggregations. *Q *values of pups were also explored in hybrid females to determine the hybridity of pups belonging to mothers that bred on particular beach types and displayed differing degrees of habitat fidelity.

## Results

### Species and hybrid spatial distributions in relation to habitat type

**Figure 2:**
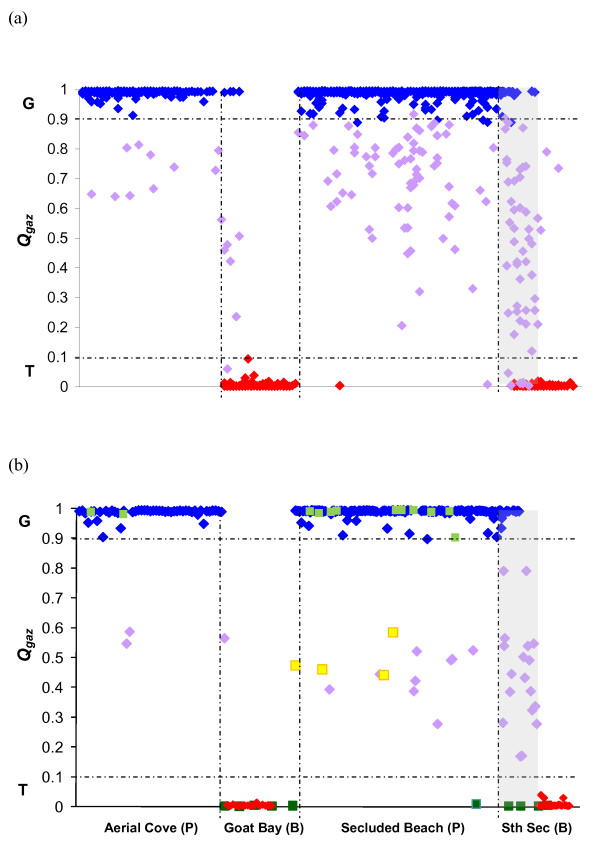
**Spatial distribution of a) pups and b) adults (squares represent males), showing strong separation of *A. gazella *(blue for females and pups, dark green for males, *Q*_*Gaz *_≥ 0.9) and *A. tropicalis *(red for females and pups, light green for males, *Q*_*Gaz *_≤ 0.1) across the four main breeding aggregations, with habitat type (P = pebbles, B = boulders) in parentheses**. The transition zone between habitat types is shaded and illustrates the relatively high density of hybrid pups and females (purple for females and pups, yellow for males). The hybrid threshold is bounded by a horizontal, dashed line and includes all pups with *Q*_*Gaz *_between 0.1 and 0.9).

Using territory locations and species information for *A. gazella, A. tropicalis *and hybrid pups and adults from 1992-2003 (Table 1), four main breeding aggregations were identified: Aerial Cove, southern Goat Bay, Secluded Beach and southern Secluded Beach (Figure 1). A plot of location against *Q*_*Gaz *_revealed that each aggregation was comprised mostly of a single species, with very little overlap in the spatial distributions of *A. gazella *and *A. tropicalis *adults (Figure 2). One breeding territory was formed over multiple years in the transition zone between habitat types (from pebbles to boulders) at southern Secluded Beach. It was the only location on the island where adults of the two species occurred in the same territory and accordingly, contained the highest concentration of hybrid pups, up to 75% of which had genetic characteristics of F_1 _hybrids (*Q *values of 0.22 to 0.78, Figure 2).

Consistent with observations of other sympatric and allopatric breeding colonies of *A. gazella *and *A. tropicalis *[16,19,28], the distribution of parental species on Macquarie Island was closely related to habitat type. Ninety-six percent (176/183) of *A. gazella *females and all *A. gazella *males bred on open, pebbled beaches (Aerial Cove, Secluded Beach), while all *A. tropicalis *females and 90% (9/10) of *A. tropicalis *males bred in bouldered coves (Goat Bay, southern Secluded Beach, Figure 1). Over two thirds (73%) of hybrid females bred consistently on a single habitat type across years (16/22) but of these, eight bred on pebbles and eight on boulders. There was no strong evidence that this pattern was dictated by matrilineal background. While all eight hybrids that bred consistently on pebbles had mtDNA profiles of *A. gazella*, only two that bred on boulders had *A. tropicalis *mtDNA profiles, with the remainder having *A. gazella *profiles. Based on microsatellite profiles however, hybrid females that bred on pebbles had a higher mean *Q*_*Gaz *_(were genetically more *A. gazella*-like) than those that bred on boulders. This difference approached significance (t-test: t = 2.06, d.f. = 14, *p *= 0.059). The remaining hybrid females (n = 6, 27%) displayed no consistent habitat use, moving between pebbled and boulder beaches among years.

### Inter-annual site fidelity in pure species and hybrids

**Figure 3:**
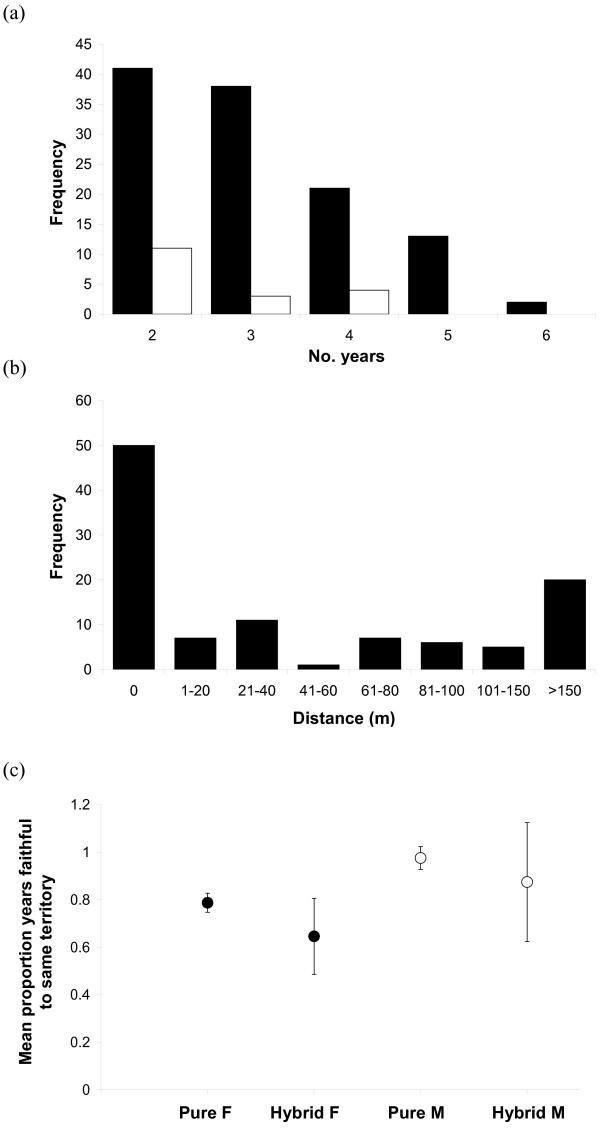
**Site fidelity of breeding adults, showing frequency histograms of the a) number of years males (white) and females (black) were observed breeding, b) distance (metres) between pupping sites among years for pure-species females, and c) the mean (±2 S.E.) proportion of years pure and hybrid adults were observed returning to the same territory to breed**.

Segregation of *A. gazella *and *A. tropicalis *may be reinforced by high inter-annual site fidelity exhibited by breeding adults of both species. Breeding locations of tagged *A. gazella *females (n = 95) and *A. tropicalis *females (n = 12) were recorded over a mean of 3.2 and 3.1 years respectively (range 2-6 yrs, Figure 3a). Of these 107 females, 21 (19%) moved relatively long distances among years, from one breeding bay to another (>250 m, e.g. between Aerial Cove and Secluded Beach, Goat Bay and Secluded Beach), but 45% (n = 48) of females consistently returned to the exact same breeding territory among years and a further 21% (n = 23) moved only to a neighbouring territory, usually only 7-28 m away (Figure 3b).

Territory males displayed significantly higher inter-annual site fidelity than females, with the maximum distance moved among years only 7 m into a neighbouring territory by one of 14 pure-species males sampled (8 *A. gazella*, 6 *A. tropicalis*, Mann-Whitney: Z = -3.27, *p *= 0.001). The remaining 13 males returned to hold the same territory over multiple years.

Hybrid territory males (n = 4) displayed a similar level of site fidelity to pure-species males. The mean proportion of years spent in a single territory (maximum number of years in one territory/number of years observed) was 0.95 for hybrid males, compared to 0.96 for *A. gazella *males and 1.0 for *A. tropicalis *males (Kruskall-Wallis: H = 1.60, d.f. = 2, *p *= 0.45, Figure 3c), and there was no significant difference in the distance moved among years by pure and hybrid males (Mann-Whitney: Z = -0.97, *p *= 0.65). Hybrid females (n = 8) showed significantly lower among-year fidelity to a territory than did pure species females: only 25% returned to the same location compared with 45% (G-test: G = 8.81, d.f. = 1, *p *= 0.005). This was also reflected in the mean proportion of years spent in a single territory, which was 0.65 for hybrid females, compared with 0.78 and 0.84 for *A. gazella *and *A. tropicalis *females respectively, although this difference was not statistically significant (Kruskall-Wallis: H = 1.51, d.f. = 2, *p *= 0.47, Figure 3). Despite this apparent lower faithfulness to a territory, the mean average distance moved by hybrid females among years was not significantly higher than that of pure females (Mann Whitney: Z: -0.45, *p *= 0.65).

### Reproductive consequences of differing habitat fidelity for pure and hybrid females

Although over half (55%) of *A. gazella *and *A. tropicalis *females changed their breeding locations among years, they were extremely faithful to the habitat type typically favoured by their species. Only five out of 107 (4.7%) pure-species females that changed their breeding location among years moved onto their atypical habitat type. The genetic characteristics of the offspring (n = 8) of the five pure *A. gazella *females that bred on boulder beaches (in heterospecific aggregations) were examined. All pups born to the five females were hybrids, as they had *Q *values < 0.9 for belonging to either of the two parental species (mean *Q*_*Gaz *_= 0.61). The purity of these pups compared to pups of *A. gazella *females that bred in conspecific aggregations (n = 183) was significantly lower (mean *Q*_*Gaz *_of pups born to females in conspecific aggregations = 0.96, Mann-Whitney: Z = -4.63, *p *< 0.0001). Production of hybrid offspring by pure females represents a major consequence of breeding in heterospecific aggregations. All hybrid offspring had *Q *values within the expected range of first generation (F_1_) hybrids produced by mating between *A. gazella *females and *A. tropicalis *males.

Hybrid females with no consistent habitat use were likely to produce various classes of hybrid pups (ie. backcrosses to both parental species). Pups born to hybrid females that displayed low habitat fidelity among years (n = 10) had greater variance in their species composition (*Q*_*Gaz*_) than those born to hybrid females that displayed high habitat fidelity (n = 13, Levene's test: F = 5.61, *p *= 0.05).

## Discussion

Identifying factors that regulate gene flow in highly mobile species experiencing hybridisation is important for understanding and predicting the outcomes of ongoing secondary contact. Using behavioural and genetic information collected over more than a decade, we explored the spatial distribution of two species of fur seal that breed over a very small spatial scale, at times only tens of metres apart and largely within sight of one another. We found almost complete segregation of the two species, which corresponded to different habitat types. High fine-scale site fidelity was exhibited by both sexes and is likely to contribute to the maintenance of this spatial segregation. Finally, we identified a potential fitness cost for pure females of breeding in heterospecific aggregations: the production of hybrid offspring.

Habitat type has been identified as an important driver of community dynamics, social system variation and spatial population structure in burrowing mammals such as rabbits, *Oryctolagus cuniculus*, southern hairy-nosed wombats, *Lasiorhinus latifrons*, and molerats, *Bathyergidae *spp. [33-35]. It has also been shown to contribute to the maintenance of discrete gene pools in a fire-bellied toad hybrid zone (*Bombina *spp, [36]). While the dominant force likely to be driving the segregation of *A. gazella *and *A. tropicalis *on Macquarie Island is a preference for members of the same species to associate, habitat type was found to further predict where species locate, consistent with other sympatric and allopatric populations of the two species [16,28,37]. There may be several explanations for this. Firstly, the two species may have become differently adapted to particular habitats in allopatry [3]. Alternatively, historical contact between the two species may have led to the evolution of different habitat preferences to avoid hybridisation. As pre-sealing distributions of the two species are unknown, historical sympatry cannot be discounted, although genetic data do not show evidence of past introgression [18,38]. Another possibility is that both species prefer the same habitat but there is competitive exclusion of one by the other. At Macquarie Island this does not seem likely, as males of the two species are of very similar sizes, are rarely seen actively competing for territories, and display the same patterns of habitat use as those in allopatric colonies. A more likely explanation is that beach type correlates with some other aspect of the physical environment that *A. gazella *and *A. tropicalis *prefer, such as access to water, slope, level of wave action, shade or exposure. For example, at Gough Island, *A. tropicalis *is thought to breed preferentially on boulder beaches that are exposed to prevailing winds and sea spray to aid in thermoregulation [37]. Unlike other sympatric species that are segregated by habitat type as a result of strong local selection (e.g. crickets, *Grillus *spp., and lizards, *Sceloporus *spp. [39,40], fur seals at Macquarie Island are highly mobile, capable of long distance migration, and not limited by the availability of both habitat types. Given that they are largely unconstrained in their initial selection of a beach location, the segregation we observed most likely reflects a behavioural preference resulting from one or a combination of the factors discussed above.

As well as consistent habitat use, high, fine-scale, inter-annual site fidelity was exhibited by breeding adults of both species. Although habitat preference and site preference are undoubtedly linked, we analysed both traits on an individual basis to decipher whether preference for a particular habitat was simply a reflection of preference for a location. Our findings suggest that while there is a high degree of site fidelity exhibited by both male and female pure-species fur seals, patterns of habitat faithfulness were also strong and importantly, independent of site fidelity for some females. Despite the finding that over half of all females changed their breeding location among years (n = 59), only five females actually moved onto their atypical beach type. The remaining females moved sites (and often bays) but still selected the beach type typically used by their conspecifics. Given that this pattern of habitat use occurs in other populations of the two species [18,21,30], our finding is not surprising, but it does demonstrate that habitat type (or the feature(s) it correlates with) is an important and consistent feature in the selection of breeding sites by *A. gazella *and *A. tropicalis*.

Individual site fidelity has been recognised as important for social and genetic structuring in colonially breeding mammals and birds (e.g. [41-43]) and has been observed in pinnipeds (e.g., grey seals, northern fur seals, Hooker's sea lions and Galapagos sea lions [30,31,44,45]). At Macquarie Island, as well as its implications for the evolution of complex social dynamics, the trait is likely to create a stable population structure over time and assist in maintaining species segregation. Our finding that among-year site fidelity was higher in territory males than breeding females has also been reported in a high-density *A. gazella *breeding colony on Bird Island, South Georgia [29]. This pattern is thought to occur because returning to the same site among years may give males a 'prior residence advantage' or enable them to avoid energetically costly combat with other males since their place in the dominance hierarchy is already established. In light of the unique species composition at Macquarie Island, high site fidelity by hybrid territory males may also have significant impacts on the spatial distribution of hybrid pups in the population. For example, in this study, two hybrid males returned to hold the same territory for four consecutive years and sired a large number of offspring. The signature of their reproductive success is apparent in the distribution of hybrid pups on the pebbled section of Secluded Beach (Figure 2). While the populations are in their post-sealing recovery phase, the polygynous nature of the fur seal mating system, coupled with a low number of successful males holding territories each breeding season may create new and significant areas of admixture in the population.

In females, faithfulness to a particular habitat (beach) type was shown to be significantly weaker in hybrids than pure-species individuals. The consequences for pure *A. gazella *females of breeding in heterospecific aggregations are potentially severe; firstly in terms of the direct reproductive costs they may incur through the production of hybrid offspring, as *A. tropicalis *pups have a lactation length two and a half times that of *A. gazella *pups (four months vs 8-10 months [11]). Additionally, if daughters display natal philopatry, as has been documented in other pinnipeds [31,45], pure-species females risk placing their offspring on the 'wrong' habitat type and perpetuating interspecific breeding encounters. Given these implications, we might expect strong selection for factors that promote segregation of the two species in order to reinforce species barriers and complete speciation [3][45]. Further work to test this would require quantification of habitat use by both species at other sympatric and allopatric colonies.

Based on the strong correlation between beach type and the distribution of both species on Macquarie Island, we may infer that one could be used to predict the other. As the population continues to expand, physical mapping of the locations and abundance of each of the habitat types at Macquarie Island could be useful for predicting potential new sites of colonisation by the two species as they continue to recover, as well as identifying areas where habitat types converge and 'hotspots' of admixture may occur. As a further step, modification of these habitats may theoretically enable the degree of hybridisation in the population to be managed. However, with both species recovering at a steady rate across their former ranges, at present there is little concern that continued hybrid production at Macquarie Island will threaten the genetic integrity of either species on a global scale. Nevertheless, this study has enabled us to identify and better understand two behavioural traits which appear to be of evolutionary significance in this instance of secondary contact.

## Conclusions

We have shown that even in a disturbed system with an initial rarity of conspecific mates and consequently a high level of hybridisation, two behavioural traits have promoted segregation of parental species and maintained the integrity of discrete gene pools. Variation in these traits has important fitness consequences for individuals, thus there may be strong selection for these traits in pure species to reduce the incidence of hybridisation. Future research will address this and related topics to further our understanding of population dynamics and the ecological and evolutionary consequences of secondary contact in these species.

## Authors' contributions

MLL examined hybridisation in fur seals at Macquarie Island for her PhD and this study forms part of her research. SDG initiated and manages the long-term fur seal monitoring program at Macquarie Island. Both PS and SDG supervised MLL during her PhD research. All authors read and approved the final manuscript.
